# Characteristics of menstrual versus non-menstrual migraine during pregnancy: a longitudinal population-based study

**DOI:** 10.1186/s10194-018-0853-3

**Published:** 2018-04-02

**Authors:** Beáta Éva Petrovski, Kjersti G. Vetvik, Christofer Lundqvist, Malin Eberhard-Gran

**Affiliations:** 1 Health Services Research Centre, Akershus University Hospital, Post Box 1000, 1478 Lørenskog, Oslo Norway; 2 Faculty of Dentistry, University of Oslo, Geitmyrsveien 69, 0455 Oslo, Norway; 3 Department of Neurology, Akershus University Hospital, Post Box 1000, 1478 Lørenskog, Oslo Norway; 4 Institute of Clinical Medicine, Campus Ahus, University of Oslo, Post Box 1000, 1478 Lørenskog, Norway; 5 Domain for Mental and Physical Health, Norwegian Institute of Public Health, Lovisenberggata 6-8, 0456 Oslo, Norway

## Abstract

**Background:**

Migraine is a common headache disorder that affects mostly women. In half of these, migraine is menstrually associated, and ranges from completely asymptomatic to frequent pain throughout pregnancy.

**Methods:**

The aim of the study was to define the pattern (frequency, intensity, analgesics use) of migrainous headaches among women with and without menstural migraine (MM) during pregnancy, and define how hormonally-related factors affect its intensity.

**Results:**

The analysis was based upon data from 280 women, 18.6% of them having a self-reported MM. Women with MM described a higher headache intensity during early pregnancy and postpartum compared those without MM, but both groups showed improvement during the second half of pregnancy and directly after delivery. Hormonal factors and pre-menstrual syndrome had no effect upon headache frequency, but may affect headache intensity.

**Conclusions:**

Individual treatment plan is necessary for women with migrainous headaches during pregnancy, especially for those suffering highest symptoms load.

## Background

Migraine is a common headache disorder that affects approximately 12% of the world’s population [25]. Although the condition is very common in both genders, its post-pubescent prevalence is about two to three times greater among women than men [7, 25]. More than half of women who suffer from migraine self-report a menstrual association of the condition [17, 28].

Menstrual migraine (MM) is defined in the appendix of the International Classification of Headache Disorders 3 beta (ICHD 3 beta) [9] as attacks of migraine without aura occurring on day 1 ± 2 of the menstrual cycle in at least two out of three menstrual cycles.

The prevalence of MM varies from 4 to 70% according to previous studies [3, 10, 13, 21, 28]. This large variation can be explained by differences in the populations studied, ascertainment and the previous lack of uniform diagnostic criteria of menstrual migraine [28]. When the current diagnostic criteria are used, about one fifth of female migraineurs from the general population have MM.

According to previous population-based studies, the majority of female migraineurs experience an improvement in migraine symptoms during pregnancy, and a third of them even report complete remission [11, 14, 22]. The frequency of headaches and migraines decrease significantly during pregnancy, especially in the second and third trimester [6, 11, 22] and increase postpartum [22]. To date, few studies have reported the course of migraine during pregnancy specifically for women with MM [4, 14, 22, 23] and the results are diverging. Although some women experience decrease in the headache frequency or that migraines disappear during the pregnancy [11, 14, 22], some may suffer throughout pregnancy. Assessment of headache frequency has been either retrospective or, in a few cases, prospective either using headache diaries [11] or repeated questionnaires [14, 22]. While clinically recruited populations may give opportunities for addressing clinical, migraine-related characteristics, they may not be representative for migraine in the general population of pregnant women [11, 12, 14, 22].

The aim of the present study was to describe the changes in the frequency and intensity of migrainous headaches and analgesics use in female migraineurs with and without self-reported MM from a large and representative prospective birth cohort. In addition, we aimed to examine how hormonally-related factors such as pre-menstrual syndrome (PMS), menstrual pain, age at menarche, hormonal contraception, regularity of the menstrual cycle and maternal age may affect the intensity of migrainous headache during and after pregnancy.

## Methods

### Study population and study design

**Fig. 1:**
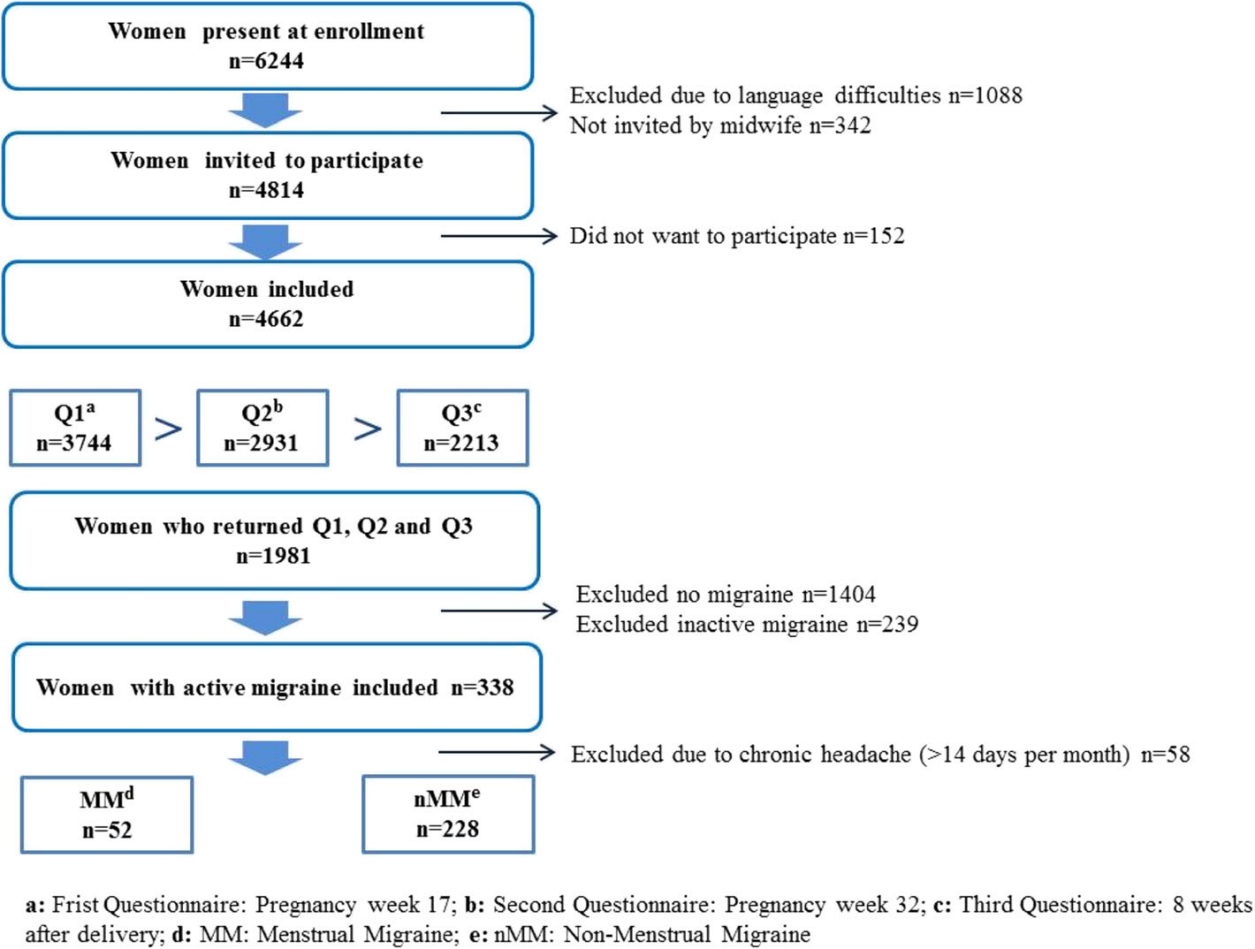
The Akershus Birth Cohort: overview of inclusion, response rates and the study sample

This study was part of the Akershus Birth Cohort (ABC) Study, which targeted all women scheduled to give birth at Akershus University Hospital, Norway. The hospital is located near Oslo, the capital of Norway, and serves a total population of approximately 400,000 individuals from both urban and rural areas; on average, 4000 women gave birth at the hospital each year during the study period. Women were recruited at the routine fetal ultrasound examination at pregnancy week 17, from November 2008 to April 2010. As part of the public health care program, this examination is free of charge to all women in the hospital’s catchment area. Pregnant women who were able to complete a questionnaire in Norwegian were eligible for the Akershus Birth Cohort to ABC. An overview of inclusion, response rates and study sample is presented in Fig. 1. Note that the sample sizes at the different time points may deviate somewhat from previous ABC publications due to small changes in the latest quality-assured data files released for research. At enrollment, 6244 women were present, of whom 1088 (17.4%) were excluded due to language difficulties and 342 (5.5%) women were not invited due to time constraints. Of the 4814 pregnant women invited to participate, 4662 (96.8%) women gave consent and were included in the study. Data were collected by self-completed questionnaires in gestational weeks 17 (Q1) and 32 (Q2), and 8 weeks after delivery (Q3). The response rates were 80.3% (3744 of 4662), 81.1% (2931 of 3613), and 79.0% (2213 of 2801) for Q1, Q2 and Q3, respectively. In total, 1981 women returned all questionnaires and comprised our baseline sample, representing 42.5% of those included. Out of the 1981 women, 338 (17.1%) had active migraine, of which 58 were excluded due to chronic headache. This resulted in a final study sample of 280 women, of which 52 (18.6%) had self-reported MM and 228 (81.4%) had non-menstrual migraine (nMM) (Fig. 1).

Women included in the study were older (full cohort: median, IQR: 30.7 (27.2–34.2) vs. study sample: median, IQR: 31.4 (28.5–34.9); *P* = 0.003), and had a higher education (full cohort: 58.8% vs. study sample: 65.4% vs.; *P* = 0.03), compared to those in the full cohort.

Additional information was obtained by linkage to the hospital’s electronic birth record. The birth record is completed by the hospital staff members and contains sociodemographic and medical information.

### Maternal characteristics

**Fig. 2:**
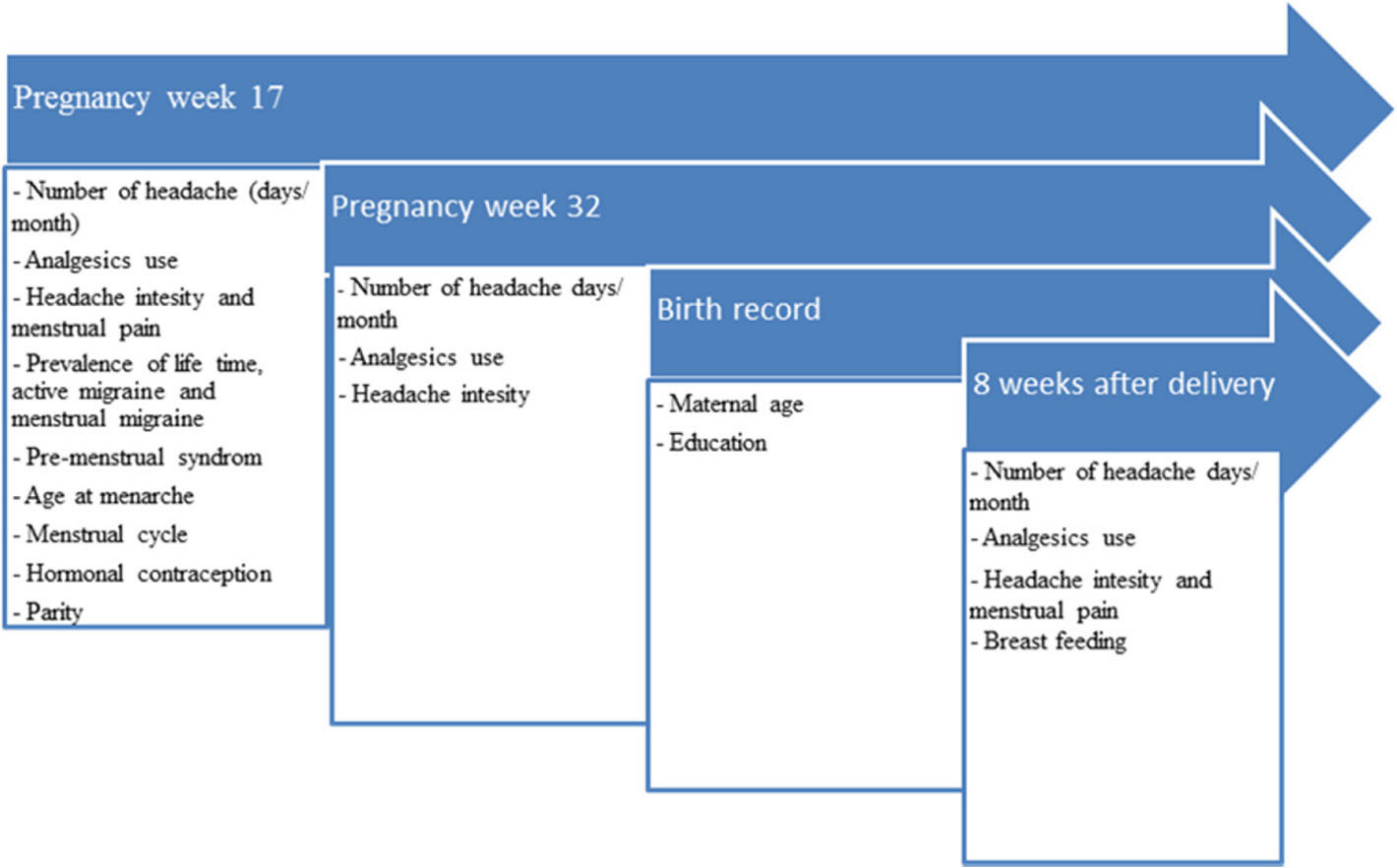
The Akershus Birth Cohort: overview of the relevant variables in the questionnaires and birth record

An overview of the relevant variables describing maternal characteristics is presented in Fig. 2.

### Migraine characteristics

The first questionnaire included specific questions about life-time (“Have you ever had migraine?”) and last year-prevalence (“Have you had migraine during the past year?”) of migraine. This enabled us to group the women into three mutually exclusive categories according to self-reported migraine status: no migraine history (“never had migraine”), previous history of migraine (“had migraine, but not during the past year”), migraine in the past year (“had migraine during the past year”) defined as “active migraine”.

Women were asked whether their migraine was menstrually-related or not. The migraine was categorized as menstrual migraine (MM), if migraine was present in at least 2/3 of menstruations [9]. The remaining cases were categorized as having nMM. Women with chronic headache, i.e. reporting more than 14 headache days per month before pregnancy, were excluded because it is impossible to judge whether their migraine attacks occurring perimenstrually occurs by chance or represents “true” menstrual migraine attacks. Information was also collected about headache frequency (days/month) in Q1, Q2, Q3 and categorized as: 0 day, 1 day/month or 2–14 days/month. Headache intensity was measured in all three questionnaires by a numeric rating scale (NRS) from 0 (no pain) to 10 (greatest pain imaginable).

### Analgesic use

Women were specifically asked about use of drugs within seven categories – drugs for headache, migraine, non-headache pain, insomnia, anxiety, depression and other psychotropic medications. For each medication group, the women could tick yes or no as to whether she used a drug, and fill in the name of medication. The three questionnaires covered different periods of use – including the preceding three months before pregnancy and beginning of pregnancy until week 17 (Q1), week 17 to 32 (Q2), and the last part of pregnancy from pregnancy week 33 until 8 weeks postpartum (Q3). In the current study, use of analgesics for headache and migraine medication were combined and categorized into two categories: ‘no headache analgesics use’ or ‘headache analgesics use’.

### Other factors

Questions related to menstrual cycles and other menstrually related symptoms were asked: regularity of menstrual cycle before pregnancy (irregular/normal), menstrual pain (NRS 0–10) and age of menarche. PMS was assessed through questions of whether the participants experienced depression before menstruation (no/yes) and if it disappeared once the menstruation began (no/yes). Those women were considered as having PMS, who gave positive answer to both questions. In addition, information about hormonal contraception before pregnancy (no/yes) and breastfeeding after pregnancy (no/yes) was collected.

Included socio-demographic and life-style characteristics and some other factors that may influence migraine were as follows: maternal age, education (primary/secondary school vs. higher education) and parity (nulliparous versus multiparous).

### Statistics

The analysis of the data was performed by descriptive statistical analysis; percentage distribution, mean and standard deviation (SD), median and interquartile range (IQR) are shown. The Chi-square (*χ*^2^) test was used to test differences of the distribution of categorical variables. Normality of continuous variables was tested on Q-Q-plot and by Shapiro-Wilk and Kolmogorov-Smirnov test. The Student t-test was used to compare means of continuous, numerical variables, when the normality assumption was satisfied; otherwise Mann-Whitney U test was used.

For trend analysis, a Generalized Estimating Equations (GEE) were used. The correlation structure was assumed to be of an ‘AR1’ type - more details about the GEE analysis has been published before [26]. The results of the analysis regarding the relationship between pain intensity (NRS) during the pregnancy and MM, are presented as unadjusted and adjusted regression coefficients (β coefficient) and with 95% Confidence Intervals (CIs). Data are adjusted for parity, hormonal contraception and education, since education and parity were significantly associated with menstrual pain, while hormonal contraception was associated with irregularity of the menstrual cycle. The PMT and the age of menarche were not included into the full model, because none of these factors were associated with the factors included into the GEE analysis. Age was included as explanatory variable. Data analysis was based on the data of 263 participants. The effect of breast feeding on the MM and intensity of pain due to migrainous headache were analyzed separately in the last period of the study (8. weeks after delivery). Multicollinearity was detected by variance inflation factor analysis (VIF).

Significance limit was set as *P* < 0.05. All statistical analyses were performed by using the Statistical Package for STATA (Stata version 14.0; College Station, TX, USA).

## Results

### Characteristics of the study sample

Pregnancy-related and demographic characteristics of the studied population are shown in Table 1. There were no significant differences according to the studied factors between the two groups (MM, nMM). The average age of the participants was approximately 31 years (mean ± SD: 31.6 ± 4.6) and most of them had a higher education (65.4%). Few (8.3%) reported menstrual irregularities before pregnancy, while approximately 12% (12.2%) had used hormonal contraception before pregnancy. The occurrence of PMS was 25.0% among women and it did not differ between the MM and nMM groups (Table 1).

**Table 1:** Characteristics of the sample according to whether the participants had menstrual-related migraine or not

	MM *N* = 52	nMM *N* = 228	*P*-value
Parity			
Nulliparous	27 (51.9%)	134 (58.8%)	0.367
Multiparous	25 (48.1%)	94 (41.2%)	
Education^a^			
Primary/secondary school	15 (32.6%)	77 (35.0%)	0.756
Higher education	31 (67.4%)	143 (65.0%)	
Menstrual cycle^b^			
Regular	47 (90.4%)	208 (92.0%)	0.697
Irregular	5 (9.6%)	18 (8.0%)	
Hormonal contraception^c^			
No	47 (90.4%)	198 (87.2%)	0.530
Yes	5 (9.6%)	29 (12.8%)	
PMS			
No	40 (76.9%)	170 (74.6%)	0.723
Yes	12 (23.1%)	58 (25.4%)	
Breast feeding^d^			
No	7 (13.5%)	36 (15.9%)	0.657
Yes	45 (86.5%)	190 (84.1%)	
	mean ± SD		
Maternal age	32.2 ± 4.5	31.5 ± 4.6	0.281
Age of menarche	12.9 ± 1.2	12.8 ± 1.4	0.725
Menstrual pain (0–10)	3.7 ± 2.2	3.7 ± 2.3	0.901

*PMS* Pre-Menstrual Syndrom, *SD* Standard Deviation, *MM* Menstrual Migraine, *nMM* Non-Menstrual Migraine

^a^ 14 missing values

^b^ 2 missing values

^c^ 1 missing value

^d^ 2 missing values

### Frequency of headache

**Fig. 3:**
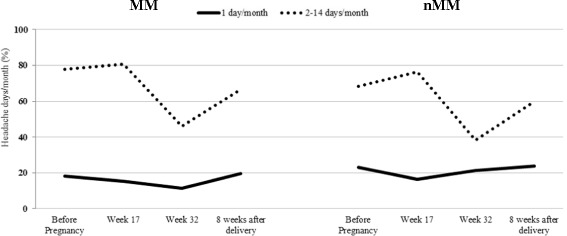
Association between menstrual migraine and headache frequency before-, during- and after pregnancy. Data shown represents that of Mentrual (MM) and Non-Menstrual Migraine (nMM) groups. Before pregnancy: 13 missing values; Week 17: 13 missing values; Week 32: 3 missing values; 8 weeks after delivery: 5 missing values

Figure 3 shows the proportion of women with headache frequency of 1 day/month and 2–14 days/month. During and after pregnancy significantly fewer women had headache frequency one day/month while the proportion having 2–14 headache days/month was higher and increased in week 17. In late pregnancy, the proportion decreased from around 80% to 40% before it increased again post-partum. Proportions of headache-free patients for MM were at pre-pregnancy, early pregnancy, late pregnancy and post-partum, respectively 4.0%, 3.8%, 42.3% and 13.7%, while headache-free patients for nMM were 8.8%, 7.1%, 40.4% and 16.5% at the same time points.

No significant differences could be detected between the MM and nMM groups. However, the number of headache days/month tended to be higher among those with MM during the whole study period than among nMM (Fig. 3).

### Frequency of analgesics use

**Fig. 4:**
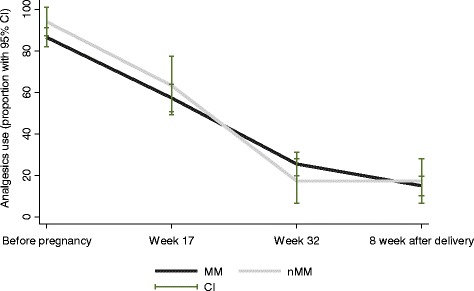
Analgesics use before, during and after pregnancy. Data shown represents that of Mentrual (MM) and Non-Menstrual Migraine (nMM) groups. Before pregnancy: 9 missing values; Week 17: 11 missing values; 8 weeks after delivery: 1 missing value; % users in each group with 95% CI: Confidence Interval. Analgesics: paracetamol, NSAIDs, opioids and triptans (used for either migraine or headache)

More than 80% (87.8%) of the participants used analgesics before pregnancy and this proportion decreased during pregnancy. However, more than 50% of the participants (58.4%) used analgesics up to week 17, and over 20% (23.9%) up to week 32. Analgesics use then stabilized 8 weeks after birth. There was no significant difference between MM and nMM during pregnancy with regard to analgesics use. A tendency towards a more drastic fall in analgesics use in MM was noted (Fig. 4).

### Migrainous headache intensity

**Fig. 5:**
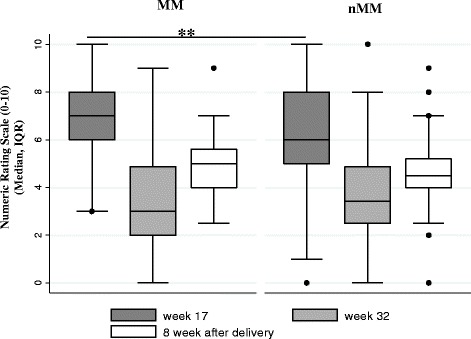
Headache intensity before, during and after pregnancy. Data shown represents that of Mentrual (MM) and Non-Menstrual Migraine (nMM) groups. **P < 0.01; IQR: Interquartile Range; No information about the pain intensity due to migraine was collected before pregnancy; The headache intensity showed nearly significant difference between the two groups post-partum (8 week after delivery) (*P* = 0.054)

The headaches intensity (NRS) showed a decreasing trend during pregnancy with an increase post-partum. Significant differences between the two groups (MM vs. nMM) could be detected on pregnancy week 17, when pain intensity appeared to be higher among those with MM (MM: median: 7 (IQR: 6–8; mean ± SD.:7.02 ± 1.75) vs. nMM (nMM: median: 6 (IQR: 5–8; mean ± SD.: 6.21 ± 1.90; *P* = 0.008). A nearly significant difference in the same direction was found also post-partum (MM: median: 5 (IQR: 4–6; mean ± SD.:4.84 ± 1.24 vs. nMM: median: 4 (IQR: 4–5; mean ± SD.: 4.53 ± 1.33; *P* = 0.054) (Fig. 5).

### Trend analysis

The results of the GEE analysis is shown in Table 2. The migraine status (nMM vs. MM), menstrual cycle regularity, menstrual pain and analgesics use were positively related to the pain intensity (NRS) in both, unadjusted and adjusted analysis. Conversely, the progressive time period during pregnancy was negatively correlated to the pain intensity in both analyses. Maternal age, parity, education and previous use of hormonal contraception showed no significant relationship with pain intensity, either in the unadjusted or in the adjusted analysis*.* While education and parity showed significant association with the menstrual pain, hormonal contraception showed association with the regularity of the menstrual cycle (data not shown).

**Table 2:** Results of the Generalized Estimating Equations (GEE) analyses

	Pain intensity Unadjusted β coefficient (95% CI)	*P- value*	Pain intensity Adjusted^a^ β coefficient (95% CI)	*P*- value
Migraine status				
nMM	Reference		Reference	
MM	0.37 (0.04–0.71)	0.027	0.45 (0.08–0.82)	0.017
Time period				
Pregnancy week 17	Reference		Reference	
Pregnancy week 32	−2.80 (−3.07- -2.53)	< 0.001	−2.61 (−2.91- -2.31)	< 0.001
8 week after delivery	−1.77 (− 2.01- -1.53)	< 0.001	−1.54 (−1.83- -1.26)	< 0.001
Menstrual pain	0.15 (0.09–0.20)	< 0.001	0.16 (0.10–0.23)	< 0.001
Menstrual cycle				
Normal	Reference		Reference	
Irregular	0.75 (0.11–1.40)	0.022	0.67 (0.05–1.29)	0.035
Analgesics use				
No	Reference		Reference	
Yes	1.26 (0.98–1.54)	< 0.001	0.48 (0.19–0.77)	0.001

*MM* Menstrual Migraine, *nMM* Non-Menstrual Migraine: ^a^Adjusted for maternal age, parity, hormonal contraception, education; *P*-value< 0.05 were considered as statistically significant

Breastfeeding showed no significant association either with the migraine status or the pain intensity due to headaches post-partum (data not shown).

## Discussion

### Main findings

We found a similar pre-pregnancy prevalence of MM among female migraineurs compared to other Scandinavian studies [13, 21, 28]. Headache frequency was high among both MM and nMM before pregnancy and during early pregnancy, while both groups improved during later parts of pregnancy with a slight worsening again during the post-partum period. The women with self-reported MM had more severe headache pain during first half of the pregnancy and directly post-partum, but headaches followed a more similar pattern during pregnancy. Other menstrual related factors such as PMS, hormonal contraception before pregnancy and age at menarche were not associated with headache intensity, while irregular menstruation was.

### Strengths and limitations

The strength of this observational study is its large, prospective sample size and representative population-based cohort of pregnant women likely to be seen and treated by GPs or obstetricians. Another strength is the use of the detailed questionnaire and that self-reported migraineurs were identified using validated questions [20].

Moreover, other, menstrual- or hormone related health issues and their fate during pregnancy could be targeted. Such phenomena could thus be compared to migraine and add a dimension not previously addressed.

The ABC study had a high response rate. For the present study, however, we required women to have responded to all three questionnaires, which represent only 42.5% of all study participants. A comparison of these women with the full cohort population indicates that the women in our sample were older and had a higher education, indicating higher socioeconomic status. The aim, however, was not to estimate the occurrence of these factors in the population, but rather the strength of associations between them. This should be considered when interpreting the results.

Nevertheless, the prevalence and migraine pattern during pregnancy described here are likely to be more representative of the general population of pregnant women than samples selected from headache clinics. We did not have the possibility of performing a clinical examination and full headache interview of the women. Also, even though the study was designed as a prospective study, information about migrainous headache before pregnancy was collected retrospectively, thus making recall bias a possibility.

All women who reported migraine occurrence in at least two out of three menstruations were classified as MM. The gold standard for diagnosing MM is a prospective headache- and menstruation diary ([9, 27]. Such a diary would have enabled a more exact evaluation of the pre-pregnancy menstrual relationship. However, this study relied upon the women’s self-report, as they were included only at week 17 of pregnancy. Thus, there is a lack of exact information about the timing of migraine attacks in relation to the first day of menstruation, and possibility for positive and negative misclassification within the two groups (nMM and MM). Furthermore, the differences between those with and without MM may have been clearer if such a specific diagnosis could have been made.

### Interpretation

The prevalence of self-reported MM among women with migraine was 18,6% in the present study, which corresponds well with the prevalence of MM in other Scandinavian studies [13, 21], and especially with a previous study from the Norwegian general population in the same age group [28].

The characteristics of migraine through pregnancy and the immediate post-partum period which we have demonstrated here also correlates well with that from previous studies [2, 6, 10, 11, 14, 15]. We demonstrate improvement for most subjects regarding the number of headache days per month, intensity of headaches, and analgesics use. The present study, however, suggested an initial increase in the proportions of women with frequent headaches during the first half of pregnancy, although this increase was slight (Fig. 3), and thus differs from most other studies. However, the same pattern has been suggested in a previous population-based Norwegian study [1]. That general population-based study did not differentiate between MM and nMM. The present study suggests a higher headache intensity for MM in the earlier parts of pregnancy and post partum but, for both types, a high headache occurrence, especially for those with more frequent headaches, among both groups during early pregnancy. Due to the lack of detailed clinical descriptors, we cannot know whether all headaches responsible for this apparent initial increased frequency were all migrainous headaches, or if tension headaches dominated some days. Our findings of a high headache intensity during this period seem to suggest a dominating migrainous headache. No significant difference could be detected regarding the frequency of migrainous headaches during the study period between the two studied groups. A recent systematic review penetrates the literature on the presence of various headaches during pregnancy, including migraine in general, and emphasizes the importance of differential diagnosis of pregnancy headache, but does not address menstrual and non-menstrual type specifically [16]**.**

The intensity of migrainous headaches showed a decreasing tendency during pregnancy, with a small post-partum increase, which is in line with the results from the GEE analysis. Three recent studies have described the characteristics of migrainous headaches during pregnancy and childbirth prospectively [2, 11, 15]. Melhado et al. and Allais et al described a subjective change in characteristics (improved, unchanged or worse) [15], while the diary study of Kvisvik et al described characteristics only from mid pregnancy onwards and headache intensity only during the post-partum period [11]. The pattern from these studies, however, seem to support our data with a define improvement only during later parts of pregnancy [2, 15] and a worsening again post-partum [11].

MM was positively associated with headache intensity during pregnancy, which contrasts with previous studies [4, 14, 19] There was also a positive association between pain intensity during pregnancy and self-reported menstrual pain before pregnancy. The latter was true even if results were adjusted for whether the migraine type is MM or nMM. This would seem to support a general effect on pain sensitivity over the menstrual cycle among some of the women which is associated with the intensity of migrainous headache during pregnancy. Such a general pain sensitivity mechanism affecting both migraine presumed to be hormonally related (MM) and migraines which are less hormonally influenced (nMM), would be expected to give a similar change pattern during pregnancy with other headaches such as tension type headache, as has been shown in other studies [2, 15]. The mechanisms behind such an association may be hormonally independent but remain to be explored.

According to the systematic review published by the World Health Organization (WHO, 2004) and data from some developing countries, the prevalence of irregular menstrual cycles was between 5 and 16% in the general population. Spierings and Padamsee et al. also found similar results; the prevalence of irregular menstrual cycles occurred in 16.7% of the migraine population [24], which depended on the definition used for irregular menstrual cycle. The latter study fits partly with our results, though we found a lower prevalence of irregular menstrual cycles of about 8% (47% of these women had received hormonal contraception). Though our results show irregular menstrual cycles have no significant association with MM (data not shown), the menstrual cycle was strongly associated with pain intensity due to headaches even after adjustment for hormonal contraception (Table 2). This suggests that other factors, related to the irregular menstrual cycle may affect pain intensity due to headache [18, 24].

Dean et al. reported a prevalence of PMS of 19–30% [5], which is in line with our prevalence of PMS being 25% in the studied population. In our analysis, no association could be detected between the migraine status and PMS.

Despite the importance of medication safety during pregnancy, a high proportion of women consumed analgesics due to migrainous headache during pregnancy and although this decreased during the pregnancy, it remained relatively high in the second- (60%) and third- trimesters (20%). Decreased use of headache analgesics during pregnancy may be explained either by an improvement in headache with a consequent reduced need for analgesics, or by a reduction in analgesics intake despite pain due to fear of foetal damage or based on direct advice from others. The remaining, considerable analgesics use in the early part of pregnancy may have several possible explanations. Some women may not initially have been aware of being pregnant and therefore continued their pre-pregnancy analgesic use though by at ultrasound at week 17, most would, naturally, be aware of their pregnancy status. Another possibility, which our data on headache frequency and intensity partly seems to support, is that the headache at this time point has not yet started to improve, and may for some even have worsened, leading to high requirements for analgesics. Also (or alternatively) a switch from more migraine-specific, effective medication perceived to be risky during pregnancy, to less effective, safer medications such as paracetamol could also contribute. The latter is supported by our previous data on specific analgesics use from the same material [8]. This study shows that for women afflicted by migraine during pregnancy, triptan and NSAIDs use falls drastically while paracetamol increases at the beginning of pregnancy. For women who did not experience migraine during pregnancy, this pattern was not seen [8].

## Conclusions

Women with MM reported higher headache intensity during early pregnancy and postpartum compared to women with nMM. Both groups improved significantly during the second half of pregnancy and immediately post partum. Hormonal factors and PMS did not affect frequency of headaches, but may affect the headache intensity during pregnancy in female migraineurs. We suggest that an individual treatment plan for migraines during pregnancy is needed especially for those women with the highest symptoms load.

## Abbreviations

ABC: Akershus Birth Cohort
CIs: Confidence Intervals
GEE: Generalized Estimating Equations
ICHD 3 beta: International Classification of Headache Disorders 3 beta
IQR: Interquartile Range
MM: Menstrual migraine
nMM: Non-menstrual migraine
NRS: Numeric Rating Scale
PMS: Pre-menstrual syndrome
Q1: Questionnaire in gestational weeks 17
Q2: Questionnaire in gestational weeks 32
Q3: Questionnaire 8 weeks after delivery
SD: Standard Deviation
VIF: Variance Inflation Factor
WHO: World Health Organization
*χ*^2^: Chi-square test

## References

[1] Aegidius K, Zwart JA, Hagen K, Stovner L (2009). The effect of pregnancy and parity on headache prevalence: the head-HUNT study. Headache.

[2] Allais G (2013). Migraine and pregnancy: an internet survey. Neurol Sci.

[3] Couturier EG, Bomhof MA, Neven AK, van Duijn NP (2003). Menstrual migraine in a representative Dutch population sample: prevalence, disability and treatment. Cephalalgia.

[4] Cupini LM, Matteis M, Troisi E, Calabresi P, Bernardi G, Silvestrini M (1995). Sex-hormone-related events in migrainous females. A clinical comparative study between migraine with aura and migraine without aura. Cephalalgia.

[5] Dean BB, Borenstein JE, Knight K, Yonkers K (2006). Evaluating the criteria used for identification of PMS. J Women's Health.

[6] Digre KB (2013). Headaches during pregnancy. Clin Obstet Gynecol.

[7] Disease GBD, Injury I, Prevalence C (2017). Global, regional, and national incidence, prevalence, and years lived with disability for 328 diseases and injuries for 195 countries, 1990-2016: a systematic analysis for the global burden of disease study 2016. Lancet.

[8] Harris GE, Wood M, Eberhard-Gran M, Lundqvist C, Nordeng H (2017). Patterns and predictors of analgesic use in pregnancy: a longitudinal drug utilization study with special focus on women with migraine. BMC Pregnancy Childbirth.

[9] Headache Classification Committee of the International Headache S (2013). The international classification of headache disorders, 3rd edition (beta version). Cephalalgia.

[10] Karli N (2012). Impact of sex hormonal changes on tension-type headache and migraine: a cross-sectional population-based survey in 2,600 women. J Headache Pain.

[11] Kvisvik EV, Stovner LJ, Helde G, Bovim G, Linde M (2011). Headache and migraine during pregnancy and puerperium: the MIGRA-study. J Headache Pain.

[12] Marcus DA, Scharff L, Turk D (1999). Longitudinal prospective study of headache during pregnancy and postpartum. Headache.

[13] Mattsson P (2003). Hormonal factors in migraine: a population-based study of women aged 40 to 74 years. Headache.

[14] Melhado E, Maciel JA, Jr., Guerreiro CA (2005) Headaches during pregnancy in women with a prior history of menstrual headaches Arquivos de neuro-psiquiatria 63:934–940 doi:/S0004-282X2005000600006 10.1590/s0004-282x2005000600006 16400408

[15] Melhado EM, Maciel JA, Guerreiro CA (2007). Headache during gestation: evaluation of 1101 women. J Can Sci Neurol.

[16] Negro A (2017). Headache and pregnancy: a systematic review. J Headache Pain.

[17] Pavlovic JM, Stewart WF, Bruce CA, Gorman JA, Sun H, Buse DC, Lipton RB (2015). Burden of migraine related to menses: results from the AMPP study. J Headache Pain.

[18] Popat VB, Prodanov T, Calis KA, Nelson LM (2008). The menstrual cycle: a biological marker of general health in adolescents. Ann N Y Acad Sci.

[19] Rasmussen BK (1993). Migraine and tension-type headache in a general population: precipitating factors, female hormones, sleep pattern and relation to lifestyle. Pain.

[20] Rasmussen BK, Jensen R, Olesen J (1991). Questionnaire versus clinical interview in the diagnosis of headache. Headache.

[21] Russell MB, Rasmussen BK, Thorvaldsen P, Olesen J (1995). Prevalence and sex-ratio of the subtypes of migraine. Int J Epidemiol.

[22] Sances G, Granella F, Nappi RE, Fignon A, Ghiotto N, Polatti F, Nappi G (2003). Course of migraine during pregnancy and postpartum: a prospective study. Cephalalgia.

[23] Serva WA (2011). Course of migraine during pregnancy among migraine sufferers before pregnancy. Arq Neuropsiquiatr.

[24] Spierings EL, Padamsee A (2015). Menstrual-cycle and menstruation disorders in episodic vs chronic migraine: an exploratory study. Pain Med.

[25] Stovner L (2007). The global burden of headache: a documentation of headache prevalence and disability worldwide. Cephalalgia.

[26] Verbeke G, Fieuws S, Molenberghs G, Davidian M (2014). The analysis of multivariate longitudinal data: a review. Stat Methods Med Res.

[27] Vetvik KG, Benth JS, MacGregor EA, Lundqvist C, Russell MB (2015). Menstrual versus non-menstrual attacks of migraine without aura in women with and without menstrual migraine. Cephalalgia.

[28] Vetvik KG, Macgregor EA, Lundqvist C, Russell MB (2014). Prevalence of menstrual migraine: a population-based study. Cephalalgia.

